# Bar Adsorptive Microextraction Approach for Trace Determination of Local Anesthetics in Urine Matrices

**DOI:** 10.3390/molecules30010068

**Published:** 2024-12-27

**Authors:** Joana R. P. Pereira, Daniela C. Rocha, Nuno R. Neng, Paulo Maurício, M. Edite Torres, Samir M. Ahmad, Alexandre Quintas

**Affiliations:** 1 Laboratório de Ciências Forenses e Psicológicas Egas Moniz, Molecular Pathology and Forensic Biochemistry Laboratory, Egas Moniz Center for Interdisciplinary Research, Egas Moniz School of Health and Science, Quinta da Granja, 2829-511 Almada, Portugal; jrpereira@egasmoniz.edu.pt (J.R.P.P.); etorres@egasmoniz.edu.pt (M.E.T.);; 2 Centro de Química Estrutural, Institute of Molecular Sciences, Departamento de Química e Bioquímica, Faculdade de Ciências, Universidade de Lisboa, Campo Grande, 1749-016 Lisboa, Portugal; 3 Oral Rehabilitation Department, CiiEM, Instituto Universitário Egas Moniz, Quinta da Granja, Monte de Caparica, 2829-511 Caparica, Portugal

**Keywords:** local anesthetics, urine matrices, bar adsorptive microextraction (BAµE), solid phase microextraction (SPME) LC Tips, GC-MS

## Abstract

The present work reports the development, optimization, and validation, of a methodology to determine lidocaine, procaine, tetracaine, and benzocaine in urine matrices. Two extractive preconcentration techniques, solid-phase microextraction (SPME) LC Tips and bar adsorptive microextraction (BAμE), were studied and applied to the four target anesthetics, followed by gas chromatography-mass spectrometry (GC-MS) analysis. Several parameters that could affect microextraction and back-extraction were optimized using two different designs of experiments (Box–Behnken and full-factorial) to maximize extraction efficiency from aqueous media. Under optimized experimental conditions, the BAμE technique showed better performance than SPME LC Tips and was chosen for validation assays and urine sample analysis. In blank urine, the BAµE/GC-MS methodology revealed suitable sensitivity (LOD between 2 and 18 ng/mL), good linearity (*r*^2^ ≥ 0.9945) between 0.5 and 30.0 µg/mL and recovery yields of 30.3–97.9%. Good precision (%RSD ≤ 8.8%) and accuracy (bias % between −15.9 and 15.0%) values were achieved. The developed methodology was successfully applied to the target anesthetics analysis of volunteers’ urine matrices and proved to be an environmentally friendly alternative to monitor trace levels of local anesthetics in complex matrices compared to other extraction techniques.

## 1. Introduction

Local anesthetics are commonly used in medical and dental treatments [1]. However, due to their stimulant properties in the central nervous system (CNS), they are often used as adulterants of cocaine, thus there is also a forensic purpose in developing an analytical method for these substances. Local anesthetic use as a cocaine adulterant occurs because they are readily available, relatively cheap, and can mimic and even enhance the taste and effects of cocaine, masking the dilution process and the product’s poor quality [2,3,4]. More importantly, these local anesthetics potentiate the effects of cocaine and can lead to the development of unexpected and more harmful side effects, increasing the risk of an overdose. Currently, the local anesthetics most used as cocaine adulterants are lidocaine, procaine, benzocaine, and tetracaine [3]. Furthermore, there are also reports of local anesthetics (i.e., lidocaine) used as a substance of abuse, and there are reports of death due to its consumption [5,6]. In general, these local anesthetics exhibit toxicity in the central nervous system and in the cardiovascular system. Individuals exposed to local anesthetics exhibit symptoms of CNS depression, manifesting confusion and seizures, cardiac complications such as hypotension, arrhythmia, and, in extreme cases, cardiorespiratory arrest and death [1,3,7]. In the case of benzocaine, it can also trigger methemoglobinemia, a condition that reduces the blood’s ability to carry oxygen, resulting in cyanosis, hypoxia, and dyspnea [8].

Local anesthetics are mainly metabolized in the liver or plasma [9,10]. Generally, about 5% of the parent compound is excreted unmetabolized in urine. Hence, sample preparation and enrichment are performed before analysis, usually by chromatography coupled with mass spectrometry (MS). Liquid–liquid extraction (LLE) and solid-phase extraction (SPE) are commonly used as sample preparation methods for the extraction of local anesthetics from biological samples [10,11]. However, in the past decades, there has been concern about making sample preparation more straightforward and eco-friendly. As such, approaches have included miniaturization, simplification, higher selectivity and sensitivity, elimination of toxic and hazardous organic solvents, and reduced sample volume [12,13]. All these improved attributes are congruent with the green analytical chemistry principles [14].

Recent sample preparation techniques that have already been applied to the analysis of local anesthetics include SPME, liquid–liquid microextraction (LLME), microextraction by packed sorbent (MEPS) and membrane extraction [10]. Nevertheless, two examples of modern and recently developed sample extraction techniques that, to the best of our knowledge, have not been used yet for the extraction of local anesthetics are SPME LC Tips and BAµE. SPME LC Tips consist of SPME fibers coated with HPLC-type silica particles held in place by a polymeric binder and are suitable for immersion and extraction in biological fluids and solvent desorption. The advantages of these fibers are their simplicity, ease of automation, minimal organic solvent consumption, and minimization of interferences [15,16]. On the other hand, BAµE is an analytical technique in which a bar-shaped polypropylene device is coated with a convenient adhesive, where sorbent materials are fixed. BAµE operates under a floating sampling technology mode, demonstrating outstanding performance for trace analysis of polar compounds (i.e., logarithm of octanol–water partition coefficients lower than three (log *K*_O/W_ < 3)) from aqueous media and biological matrices. BAµE devices are easily lab-made and allow the choice of the most convenient sorbent phase for each target analyte. Recently, novel advances were implemented by downsizing the microextraction device to half (7.5 mm long and 3 mm in diameter), thus minimizing the back-extraction solvent volume to 100 μL. With these new improvements, this technique is more environmentally friendly, easy to implement, and cost-effective, demonstrating remarkable performance with high enrichment factors. In addition, it is very sensitive and selective, showing good reproducibility and robustness, presenting negligible analytical interferences [12,13,17,18,19].

**Figure 1:**
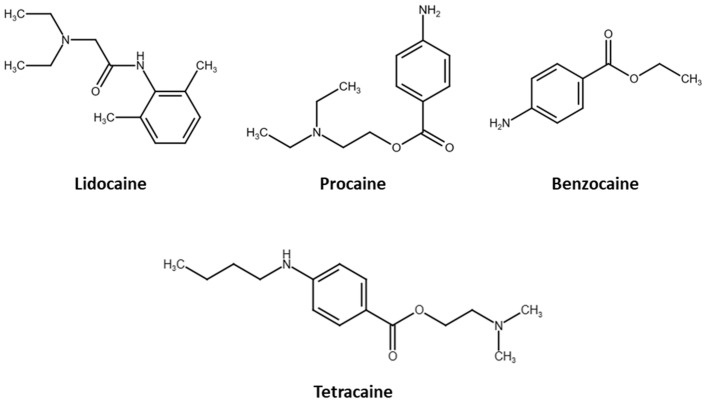
Chemical structures of the four local anesthetics studied in the present work [20].

In the present work, a new analytical approach is developed for trace determination of lidocaine, procaine, tetracaine, and benzocaine in urine matrices (Figure 1) [20]. During the development of the analytical method, two extraction and preconcentration methodologies to the target anesthetics, SPME LC Tips and BAμE, were tested, compared, and their performance and effectiveness discussed. For the identification and quantification of the target anesthetics, a gas chromatography coupled to mass spectrometry operating in the acquisition mode of selected ion monitoring (GC-MS(SIM)) method was developed, optimized, validated, and applied on human urine samples.

## 2. Results and Discussion

### 2.1. GC-MS(SIM) Optimization

The first step in this work was to establish instrumental conditions more appropriate to the local anesthetics under study. A solution containing the four target analytes was analyzed by GC-MS operating in full-scan mode acquisition to obtain each local anesthetic’s retention times and mass spectral fragmentation pattern. Based on the characteristics of the spectral data, target ions were selected (Table 1) to achieve high selectivity and sensitivity for operating in SIM acquisition mode. By monitoring the selected ions, high response and symmetrical peak shape were achieved in suitable analytical time (<10 min) under convenient chromatographic conditions.

The calibration curve showed good linearity, ranging from 5.0 to 300.0 µg/mL for lidocaine, tetracaine, and benzocaine and from 5.0 to 100.00 µg/mL for procaine. The determination coefficients (*r*^2^) were higher than 0.9981. Instrumental limit of detections (LOD) ranged from 0.03 to 0.08 μg/mL and limit of quantifications (LOQ) from 0.11 to 0.26 μg/mL, corresponding to a signal-to-noise ratio of 3:1 and 10:1, respectively. Intra- and interday precision and accuracy were also evaluated. Precision, expressed as relative standard deviation (%RSD), was below 7.0% for intraday assays and below 6.2% for interday assays. Regarding accuracy, the bias (%) range was between −18.5 and 15.0% for intraday assays and between −18.8 and 14.6% for interday assays. In addition, no carryover was observed after the injection of blank runs, for which the background observed was consistently below the LODs achieved.

### 2.2. SPME LC Tips and BAµE Optimization Assays

#### 2.2.1. Microextraction Parameters

Several parameters may affect microextraction efficiency, as this process is based on the equilibrium of the analytes between the bulk aqueous solution and the sorbent phase [17,21,22]. For this reason, parameters such as matrix pH (2.1, 7.2, and 12.3), extraction temperature (25 °C, 35 °C and 45 °C), and extraction time (5 min, 32.5 min, and 60 min), using the Box–Behnken design (BBD) were studied. This experimental design can adapt to the full quadratic model of the response surface design and is more efficient than three-level full factorial designs and central composite designs. In this design, the treatment combinations are at the midpoints of the cube’s edges and at the center. BBD needs three levels for each factor. Furthermore, BBD does not contain factors at their highest or lowest levels, avoiding experiments performed under extreme conditions for which unsatisfactory results might occur [23,24].

**Figure 2:**
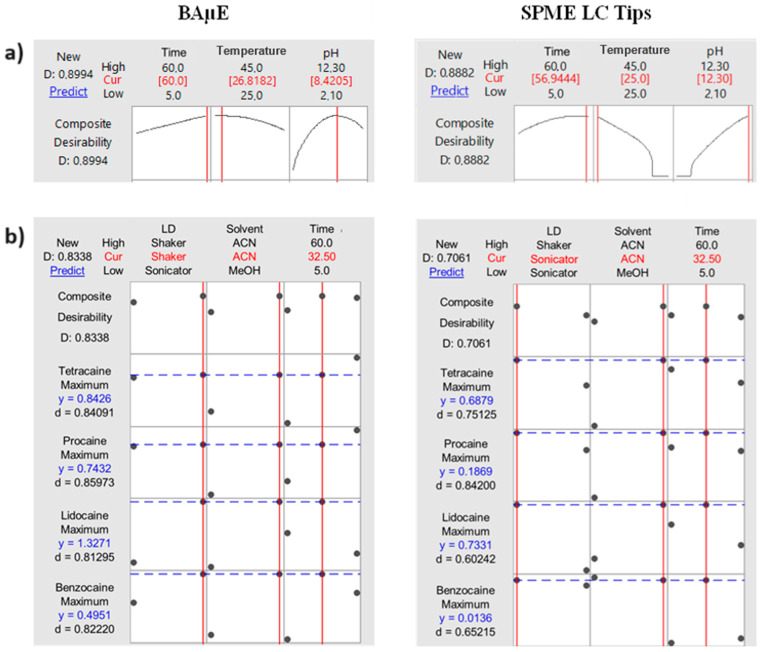
Overall BBD optimization for the extraction stage (**a**) and full factorial design optimization for the liquid desorption stage (**b**) of the procedure using BAµE devices and SPME LC Tips. y—the predicted response at the current variable settings; D—composite desirability; d—individual desirability; Cur—current variable displayed on the graphs below; LD— liquid desorption mode; time units in min and; temperature units in °C.

The results obtained (Figure 2a) demonstrate no significant differences between the optimal microextraction conditions for BAµE and SPME LC Tips. The equilibrium time and extraction temperature influence microextraction kinetics by potentially constraining the distribution of analytes between the bulk matrix and the sorbent phase, thus affecting the recovery yield [19]. In this case, the optimal microextraction time was 60 min, and the ideal temperature was 25–27 °C, for both techniques. Matrix characteristics also play an important role in the thermodynamic parameters [22]. One of these important characteristics is the matrix pH. Usually, the non-ionized form of the compounds seems to promote better recovery yields from aqueous media, as it favors the reverse-phase type interactions with the sorbent phases [25]. The results indicate that, for BAµE, the optimal pH was 8.4 and, for SPME LC Tips, the optimal pH was 12.3. Although these values are different, a pH of 12.3 was adopted for both techniques due to the satisfactory recovery yields for BAµE observed. Furthermore, from an experimental point of view, pH 12.3 is easier to prepare and keep, since that urine samples usually with acidic characteristics will be used as case studies. Additionally, according to MarvinSketch calculations [20], the pH interval where all target compounds are in the non-ionized form is between 11 and 13 which theoretically favors the extraction process.

#### 2.2.2. Back-Extraction Parameters

Some parameters, such as liquid desorption (LD) solvent and time, affect the back-extraction stage. The LD solvent must have enough strength and time to fully desorb the analytes from the sorbent phase [22]. Another factor to consider is the liquid desorption mode. Therefore, liquid desorption mode (shaking or sonication), solvents such as MeOH and ACN, as well as several desorption times (5, 32.5 and 60 min) were assayed to evaluate the back-extraction performance using a full factorial design (FFD). A FFD experiment consists of all possible combinations of levels for all factors, which means that the effect of all the factors and their interactions on the outcome is investigated. This design is advantageous when the number of design parameters is less than or equal to 4 [24,26]. The optimized results obtained for each back-extraction parameter for both techniques are shown in Figure 2b. The results in Figure 2 showed that, for both techniques, the ideal desorption solvent is ACN, and the best desorption time is 32.5 min. However, for BAµE, the ideal desorption mode is shaking, whereas for SPME LC Tips, sonication was revealed to be the ideal desorption mode. From the data obtained, the fully optimized experimental conditions for the microextraction stage were pH 12.3, 25 °C, 60 min (1400 rpm), and for the back-extraction stage were ACN as LD solvent, 32.5 min desorption time under shaking (1400 rpm) for BAµE or sonication for SPME LC Tips.

### 2.3. Comparison of Extraction Efficiencies of BAµE and SPME LC Tips

After determining the optimized conditions, the next step was to evaluate which technique had better recovery yields and extraction efficiency. The results (Table 2) in the aqueous matrix showed that BAµE had a better performance, with recovery yields between 50.8% and 100.6% (%RSD < 13.5%), while for SPME LC Tips, the recovery yields were between 1.5% and 133.7% (%RSD < 34.2%). As can be seen from the results, BAµE showed good recovery efficiencies and acceptable %RSD, unlike SPME LC Tips. For this reason, only BAµE was selected for the subsequent assays performed in the urine matrix. The recovery yields in urine slightly decreased for benzocaine and procaine, which suggest possible matrix effects. Table 2 summarizes the recovery efficiency of both methodologies under optimized conditions.

### 2.4. Validation of the Proposed Methodology

The proposed methodology (BAµE/GC-MS(SIM)) was validated using blank urine matrices. In the first approach, the analytical thresholds were determined through the LODs with values achieved between 2 and 18 ng/mL, and the respective LOQs were between 10 and 60 ng/mL. The linearity range was assessed between 0.5 and 30.0 µg/mL for lidocaine, tetracaine, and benzocaine and between 0.5 and 10.0 µg/mL for procaine (six concentration levels analyzed in triplicate). For the calibration plots, we plotted conventional linear regressions, which showed good linearity (*r*^2^ ≥ 0.9945), and according to the lack-of-fit and goodness-of-fit tests (at the confidence level 95%), they presented a good fit for the present study, i.e., the F_calc_ was consistently below the F_tab_. The results are summarized in Table S1 (Supplementary Materials). In addition, LOD, LOQ, intra- and interday accuracy and precision at three spiking levels were also estimated and summarized in Table 3.

As shown in Table 3, the precision for intraday assays (repeatability) was between 1.4% and 8.0%, and for interday assays (intermediate precision) was 2.2% to 8.8%. The intraday accuracy was between −13.6% and 13.9%, and the interday accuracy ranged from −15.9% to 15.0%. According to the Guidance for the Validation of Analytical Methodology and Calibration of Equipment used for Testing of Illicit Drugs in Seized Materials and Biological Specimens from the United Nations Office on Drugs and Crime [27], the acceptance criteria were that, for precision, the %RSD should be lower than 20% for lower concentration and better than 15% for other concentrations. For accuracy, the acceptance criteria were that the bias (%) should be within ±20% for the lower spiking level and ±15% for the remaining spiking levels. All the obtained values are in accordance with the acceptance criteria established. These validation data proved that the proposed analytical methodology presented suitable performance to determine trace levels of local anesthetics, namely lidocaine, procaine, benzocaine, and tetracaine, in urine matrices.

### 2.5. Performance Comparison with Other Microextraction Techniques

In the present work, the developed methodology (BAµE/GC-MS(SIM)) was also compared with other extraction approaches already reported in the literature [11,28,29,30,31,32] for the analysis of the target local anesthetics in urine. Table 4 summarizes the LOD, linear range, determination coefficient (*r*^2^), precision, recovery, and sample volume obtained by the proposed methodology and by other extraction-based approaches.

As can be seen, the proposed methodology includes a broader group of target compounds and has much better LOD than most of the reported methodologies, which is particularly important when performing trace analysis. Furthermore, it has a much wider linear range that may be useful in cases of substance abuse, where larger quantities of compounds are consumed, with determination coefficients similar to other works. In terms of precision, the proposed methodology gives better results than most of the compared approaches. The recovery yields are also comparable with the other methodologies. Finally, BAµE/GC-MS(SIM) requires a smaller sample volume. Therefore, the methodology proposed herein can be considered an alternative for determining the four local anesthetics (lidocaine, procaine, tetracaine, and benzocaine) in urine samples.

**Figure 3:**
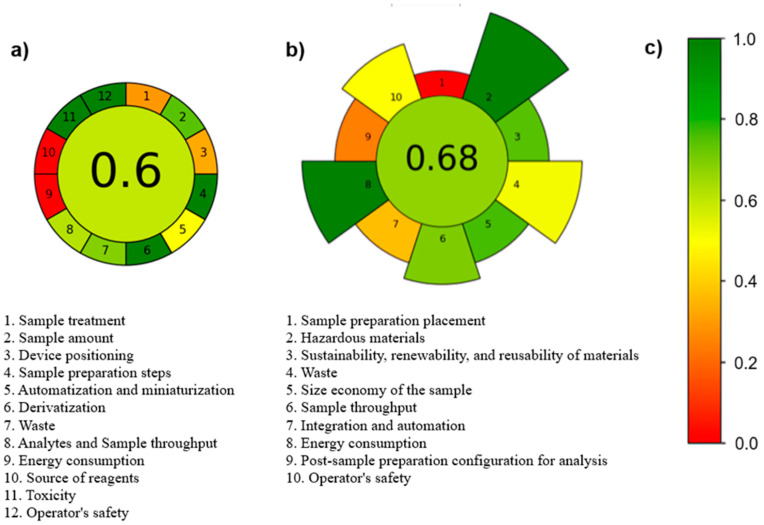
Method and sample preparation evaluation according to the analytical greenness calculator (**a**), AGREEprep calculator (**b**) and the corresponding color scale for reference (**c**).

The proposed methodology was evaluated according to the analytical greenness and AGREEprep calculator, in which all its steps are individually evaluated concerning their greenness [33,34]. The overall score is displayed at the center of the pictogram, with values near 1 and a dark green color signifying a greener procedure. The performance for each assessment criterion is represented by the color of the segment associated with its corresponding number. Considering this classification, the main limitations of the overall method are the energy consumption and the reagent sources. Regarding the sample preparation method, the main limitation is the device position as ex situ (Figure 3).

### 2.6. Application to Urine Samples

**Figure 4:**
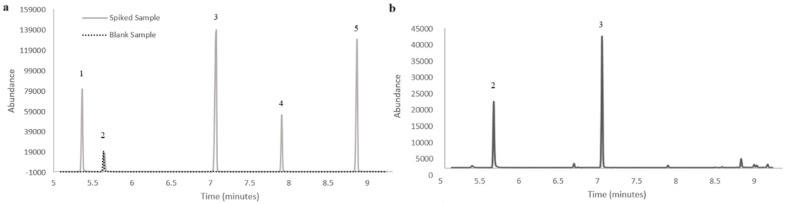
Chromatograms obtained from (**a**) spiked urine at 25.0 µg/mL (continuous line) blank urine sample without spiking (dotted line) and (**b**) a positive anonymous donor urine without spiking, analyzed through BAµE/GC-MS(SIM) methodology, under optimized experimental conditions. 1 benzocaine; 2 diphenylamine (IS); 3 lidocaine; 4 procaine; 5 tetracaine.

The feasibility of applying the present methodology to forensic work cases was tested using six urine samples from anonymous donors. The samples were analyzed using GC-MS operating in the full-scan mode acquisition, ensuring identification of the compounds. For quantification, the positive samples were analyzed in SIM mode. Figure 4 shows a chromatogram of a urine sample spiked with lidocaine, tetracaine, benzocaine and procaine and a chromatogram of a positive sample with lidocaine. The chromatograms presented show that the proposed methodology achieves good selectivity and sensitivity, with no endogenous interfering peaks at the retention times of the target compounds, including the IS. The results obtained for the urine sample analysis are shown in Table 5.

## 3. Materials and Methods

### 3.1. Chemicals, Standards and Sorbent Phases

HPLC grade methanol (MeOH, 99.9%), HPLC grade acetonitrile (ACN, 99.9%) and hydrochloric acid (HCl, 37%) were purchased from Honeywell (Charlotte, NC, USA). Sodium hydroxide (NaOH, >98.0%) was purchased from Labkem (Barcelona, Spain). Trisodium phosphate dodecahydrate (TSP, >98%) was purchased from Merck (Darmstadt, Germany). Diphenylamine (≥99%), used as internal standard (IS), procaine hydrochloride (≥97%) and benzocaine hydrochloride (≥99%) were purchased from Sigma-Aldrich (Steinheim, Germany). Tetracaine hydrochloride (>98.0%) and lidocaine hydrochloride (>99.0%) were purchased from TCI (Tokyo, Japan). Ultra-pure water was obtained from Direct-Q^®^ water purification system from Merck Millipore (Burlington, MA, USA).

Stock solutions were prepared by dissolving the referred local anesthetics standards or the diphenylamine standard in MeOH or ACN. Then, they were stored at −20 °C in amber glass vials and renewed weekly. The buffer solutions were prepared by dissolving the TSP in distilled water, adjusting with HCl or NaOH when necessary, and stored at 4 °C. The respective pH values were measured with a pH meter (MeterLab^®^ PHM210 Standard pH Meter, Terni, Italy). The sorbent phase used for coating the BAµE devices was ENVI-18 (C18, reversed phase octadecyl silica polymer; 45 µm particle size, 60 Å pore size, 475 m^2^/g surface area) and the SPME LC Tips with C18 coating were purchased from Supelco (Bellefonte, PA, USA).

### 3.2. Urine Matrices

Six urine samples were obtained from volunteers who had been anesthetized during dental care and collected after between 2 to 6 h. All samples were provided in total anonymity without any personal information about the donors. Additionally, blank urine samples were provided for the validation process by members of our investigation group who guaranteed not to have consumed any of the local anesthetics under study. Upon arrival at the laboratory, all the samples were frozen (−80 °C) until use. All the biological samples were thawed in a multi-function 3D rotator (Grant Instruments™ PS-M3D, Royston, UK) and filtered with a 0.45 µm (PTFE) syringe filter (Labfil, Zhejiang, China) before use. No other pre-treatment was carried out.

### 3.3. Experimental Set-Up

#### 3.3.1. Preparation of the BAµE Devices and Activation of SPME LC Tips

The BAµE devices were lab-made, as previously reported [12,17,21,22]. Each device was prepared by coating polyethylene hollow cylindrical tubes (7.5 mm long and 3 mm in diameter) with powdered C18 sorbent phase (0.5 to 2.5 mg) using adhesive films. After being produced, these devices were stored at room temperature in closed glass flasks. Before extraction to remove potential impurities and activate the devices, BAµE bars and commercial SPME LC Tips were cleaned with 1.5 mL of methanol/ultrapure water (50/50%, *v*/*v*) with 1400 rpm shaking at 25 °C for 20 min, using a thermo-shaker (Biosan TS-100, Riga, Latvia).

#### 3.3.2. SPME LC Tips and BAµE Optimization Assays

**Figure 5:**
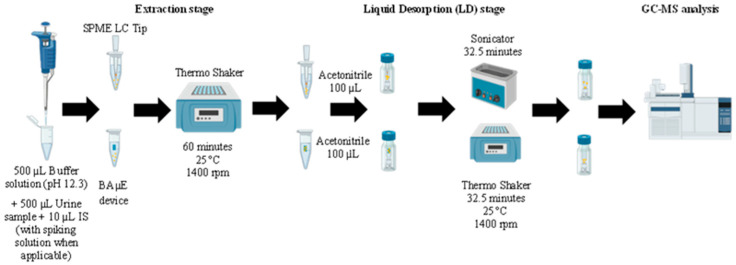
Experimental scheme of the proposed methodologies. Created with BioRender.com.

To identify the ideal conditions for extracting and analyzing the local anesthetics, we performed optimization assays for both techniques (SPME LC Tips and BAµE). In these assays, 1 mL of TSP buffer was added to a 2 mL Eppendorf tube and spiked with 10 µL of a mixed solution of all four anesthetics (50 µg/mL) and IS (100 µg/mL). Then, a BAµE device or a SPME LC Tip was introduced into that same tube. The microextraction process was performed by shaking at 1400 rpm using a thermos-shaker. After microextraction, the devices were removed from the tube. For the back-extraction stage, they were placed into a glass vial with inserts having 100 µL of a solution of organic solvent followed by shaking (1400 rpm) or ultrasonic treatment (TPC Advance Dentsonic Ultrasonic Cleaner, City of Industry, CA, USA) at 25 °C. After the back-extraction stage, the devices were removed from the insert before sealing the vial and proceeding to the instrumental analysis. Several parameters were studied, following experimental design approaches, and evaluated with Minitab^®^ Statistical Software, LLC (version 17.1.0). Initially, the microextraction process was optimized through Box–Behnken design. The three factors selected as independent variables were extraction temperature (X1), matrix pH (X2), and extraction time (X3), with three levels each: X1: 25 °C, 35 °C, 45 °C; X2: 2.1, 7.2, 12.3; and X3: 5 min, 32.5 min, 60 min. The back-extraction stage was optimized through full factorial design. The factors selected as independent variables were liquid desorption mode (X1: shaking or ultrasonication), extraction solvent (X2: MeOH or ACN), and liquid desorption time (X3: 5 min, 32.5 min or 60 min). The response was the ratio of compound chromatographic peak area and IS chromatographic peak area. Each experiment was performed in triplicate. Figure 5 shows the experimental scheme of the proposed methodologies.

#### 3.3.3. Validation Assays

For method validation, all the assays were performed under optimized conditions and in triplicate. In this sense, for each assay, 10 µL of local anesthetics and IS mix was added to a mixture of 0.5 mL of blank urine and 0.5 mL of TSP buffer solution. For the validation process, several parameters were evaluated, such as selectivity, analytical thresholds, linearity, accuracy and precision. To assess the selectivity, the optimized BAµE/GC-MS(SIM) method was applied to urine control samples, and the absence of interfering compounds at the studied local anesthetics retention time was verified. Calibration standards were prepared between 0.5 and 30.0 µg/mL (lidocaine, tetracaine, and benzocaine) or between 0.5 and 10.0 µg/mL (procaine) to assess the linearity (estimated with the lack-of-fit and goodness-of-fit tests) and the coefficients of determination (*r*^2^). In order to determine intra- and interday accuracy and precision, several assays were performed using different spiking concentrations of local anesthetics standard solutions, including 1.5, 15.0, and 24.0 µg/mL (for lidocaine, tetracaine, and benzocaine) or 1.5, 5.0, and 8.0 µg/mL (for procaine) in urine, corresponding to low, medium, and high concentrations, respectively. Interday assays were performed on three consecutive days, and intraday assays were performed on the same day.

#### 3.3.4. Urine Sample Assays

After the optimization and validation stages, the developed methodology was applied to the analysis of human urine samples donated by volunteers. The assays were performed as in the validation, under optimized conditions, but without spiking. To 0.5 mL of urine, 0.5 mL of buffer, and 10 µL of IS solution (100 µg/mL) were added prior to microextraction.

#### 3.3.5. Instrumental Set-Up

GC-MS analyses were performed with an Agilent Technologies system (USA) constituted by a 6890 Network Gas Chromatograph System, equipped with an Agilent 7683 autosampler, coupled to an Agilent 5973 Network Mass Selective Detector. All data were recorded, and instrumental control was performed using MS ChemStation software (E.02.00.493). The injections volume was 2 µL in split mode (pressure 16.18 psi, split ratio 2:1, split flow 3.0 mL/min), with the injector at 280 °C. A capillary column Mega 5MS (30.0 m × 0.25 mm × 0.25 µm; 5% phenyl, 95% methylpolysiloxane,–Mega S.r.l., Legnano, Italy) was used for the GC analysis, along with helium as the carrier gas, in constant flow of 1.5 mL/min. In full-scan mode, oven temperature was programmed to start at 100 °C and then increase to 300 °C (20 °C/min), achieving a total run time of approximately 10 min. In the selected ion monitoring (SIM) mode, oven temperature was programmed to start at 100 °C and then increase to 285 °C (20 °C/min), achieving a total run time of approximately 9.25 min. The transfer line temperature was 280 °C, the quadrupole analyzer temperature was 150 °C, and the ion source temperature was 230 °C. A solvent delay of 5 min was selected. Electron ionization (70 eV), with an ionization current of 34.6 µA and a multiplier voltage of 1200 V, was used. In the full-scan mode, a range of masses between 55 and 550 Da was selected. In SIM mode, several groups of ions were monitored in a defined time frame according to their retention time (Table 1), maintaining a dwell time of 100 ms^−1^.

The instrumental sensitivity was checked by means of limits of detection (LOD) and quantification (LOQ). The analytical limits were estimated by injecting diluted standard mixtures of the compounds of interest and identifying the minimum amount of each compound that was still detectable, using signal-to-noise ratio of 3 for LOD and 10 for LOQ. Instrumental linearity was also addressed by preparing and injecting calibration standards of different concentrations. Intra- and interday accuracy and precision were determined as well. For this purpose, several assays with three levels of concentrations of local anesthetics were performed. Interday assays were performed on three different days, and intraday assays were performed on the same day.

## 4. Conclusions

The analytical methodology presented in this work was fully developed, optimized, validated, and applied for trace determination of lidocaine, procaine, tetracaine, and benzocaine in urine matrices. Comparing the performance and effectiveness of SPME LC Tips and BAμE to assess the preconcentration of the four target anesthetics, BAμE showed better performance. BAµE/GC-MS(SIM) methodology revealed remarkable analytical performance under optimized experimental conditions, including selectivity, accuracy, precision, suitable detection limits, excellent linear dynamic ranges, and determination coefficients. Moreover, the results show that the proposed approach compares favorably with other analytical strategies already reported in the literature. This new approach is simple, user-friendly, and cost-effective, does not require a derivatization step, and employs negligible amounts of organic solvents (100 µL) and urine samples (0.5 mL) per assay, which is in accordance with green chemistry principles. The BAµE/GC-MS(SIM) methodology has proved to be a suitable alternative for the trace analysis of local anesthetics in urine samples in general, but more importantly, in a forensic context, where analytic cost is a prominent issue.

## Figures and Tables

**Table 1:** Retention times and target ions for each local anesthetic studied by GC-MS(SIM).

Local Anesthetic/ Compound	Retention Time (min)	Ions (*m*/*z*)	Relative Intensity (%)
Benzocaine	5.30	92/**120** */137/165	22.5/100/15.0/30.0
Diphenylamine (IS)	5.60	77/115/168/**169** *	
Lidocaine	7.00	58/72/**86** */234	7.5/3.3/100/1.7
Procaine	7.90	**86** */99/120/164	100/37.5/32.0/9.0
Tetracaine	8.80	**58** */71/105/176	100/77.5/13.0/14.5

* Ion for quantification; base peaks in bold; molecular ions are underlined.

**Table 2:** Recovery yields (and respective %RSD values) obtained for each target local anesthetic in aqueous matrix when utilizing BAμE or SPME LC Tips and in urine matrix utilizing BAμE.

Local Anesthetic (Spiked with 50 µg/mL)	Recovery ± RSD (%) Aqueous Matrix (n = 6)		Recovery ± RSD (%) Urine Matrix (n = 5)
	BAμE	SPME LC Tips	BAμE
Benzocaine	50.8 ± 7.4	1.5 ± 34.2	30.0 ± 7.3
Lidocaine	100.6 ± 7.4	76.8 ± 20.6	95.3 ± 1.0
Procaine	95.1 ± 9.5	38.6 ± 21.3	76.0 ± 3.1
Tetracaine	84.8 ± 13.5	133.7 ± 11.9	97.9 ± 4.0

**Table 3:** LOD, LOQ, intra- and interday recovery, accuracy and precision levels (n—number of replicates) obtained for the four local anesthetics at three different concentrations by BAµE/GC-MS(SIM) methodology, under optimized experimental conditions.

Local Anesthetic	LOD (ng/mL)	LOQ (ng/mL)	Spiking Level (µg/mL)	Intraday (n = 3)		Interday (n = 9)		
				Precision (RSD %)	Accuracy (Bias %)	Precision (RSD %)	Accuracy (Bias %)	Recovery (%)
			1.5	6.5	1.4	8.8	−4.9	43.9
Benzocaine	18	60	15.0	3.0	5.8	4.4	10.7	41.9
			24.0	8.0	3.0	7.2	7.0	44.8
			1.5	1.6	13.9	4.0	6.5	95.8
Lidocaine	2	10	15.0	1.4	13.3	2.5	15.0	93.9
			24.0	4.8	7.2	5.5	10.6	89.9
			1.5	3.4	1.5	4.7	−1.4	84.3
Procaine	4	10	5.0	1.4	8.7	3.3	12.6	82.1
			8.0	5.9	8.3	5.4	11.9	89.9
			1.5	6.0	−13.6	6.7	−15.9	94.5
Tetracaine	5	20	15.0	1.6	11.5	2.2	12.2	97.5
			24.0	4.1	7.4	5.5	9.2	98.7

**Table 4:** Comparison of the proposed methodology with other extraction techniques for the determination of local anesthetics in urine samples.

Extraction Technique	LLE	SPE	DLLME ^c^	MEPS ^d^	LPME ^e^	HFME ^f^	BAµE
Instrumental system	SSI ^a^-LC-MS	CZE-DAD ^b^	HPLC-DAD	LC-MS/MS	HPLC-UV	HPLC-UV	GC-MS
Target local anesthetics	Tetracaine Lidocaine Procaine	Lidocaine	Lidocaine	Lidocaine	Lidocaine Tetracaine	Lidocaine	Benzocaine Lidocaine Procaine Tetracaine
LOD (µg/mL)	0.38	0.11	n.a.	0.0002	0.05	0.001	0.002–0.018
Linear range (µg/mL)	0.75–12.0	0.5–10	0.18–1.5	0.001–0.47	0.1–10.0	0.5–5	0.5–30.0
Determination coefficients (*r*^2^)	≥0.992	0.997	0.9923	≥0.999	0.999	0.998	≥0.9945
Precision (%RSD)	≤12.2	≤9.1	≤14.9	≤9.4	≤5.5	≤11.7	≤8.8
Recovery (%)	60.0–85.5	≥94.9	44.62–76.63	n.a.	≥94.1	33.2	30.0–97.9 (urine matrix)
Sample volume (mL)	0.5	10	4.0	n.a.	n.a.	4	0.5
Reference	[28]	[29]	[30]	[31]	[11]	[32]	This work

^a^ Sonic spray ionization; ^b^ capillary zone electrophoresis with diode-array detection; ^c^ dispersive liquid–liquid microextraction; ^d^ microextraction by packed sorbent; ^e^ liquid-phase microextraction; ^f^ hollow fiber microextraction; n.a.—information not available.

**Table 5:** Target local anesthetics and respective concentrations present in the volunteers’ urine samples analyzed by BAµE/GC-MS(SIM) methodology, under optimized experimental conditions.

Sample	Local Anesthetics Identified	Compound Amount (µg/mL)
1	Lidocaine	2.0
2	Lidocaine	<LOQ
3	Lidocaine	0.6
4	Lidocaine	1.2
5	Lidocaine	4.1
6	No compounds identified	<LOQ

## Data Availability

Data is contained within the article or Supplementary Material.

## Supplementary Materials

The following supporting information can be downloaded at: https://www.mdpi.com/article/10.3390/molecules30010068/s1, Table S1: Calibration equations, *r*^2^, lack-of-fit and goodness-of-fit tests (at confidence level of 95%) achieved for the four local anesthetics through BAµE/GC-MS(SIM) methodology, under optimized experimental conditions.
