# Supplementary material for: Bar Adsorptive Microextraction Approach for Trace Determination of Local Anesthetics in Urine Matrices
Source: Molecules. 2024 Dec 27;30(1):68. doi: 10.3390/molecules30010068 (PMC11722190; doi:10.3390/molecules30010068)
Supplement: Supplementary file 1 [file molecules-30-00068-s001.zip › molecules-3384043-supplementary.pdf]

Supplemental Materials:

Table S1. Calibration equations, r^2, lack-of-fit and goodness-of-fit tests (at confidence level of 95 %) achieved for the four local anesthetics through BAμE/GC-MS(SIM) methodology, under optimized experimental conditions.

Local Anesthetics	LoF test		GoF test		Calibration Equation	r^2
	Fcalc	Ftab	Fcalc	Ftab		
Benzocaine	0.0028	3.7083	0.0008	19.0000	y = 0.1784x + 0.1609	0.9984
Lidocaine	0.0039	4.4590	0.0005	199.5000	y = 0.3677x + 0.5868	0.9954
Procaine	0.7849	3.2592	0.2199	9.5521	y = 0.3360x + 0.2530	0.9945
Tetracaine	0.3141	3.2592	0.1195	9.5521	y = 0.3000x + 0.5345	0.9965

Lack-of-Fit Test Fcalc mathematic formulae

$$F_{calc} = \frac{MSSLoF}{MSS\ error} = \frac{p\sum(\bar{y_i} - \hat{y_i})^2/(n-2)}{\sum(y_i - \bar{y_i})^2/n(p-1)}$$

Legend: $\bar{y_i}$ = average signal value of the replicates at concentration i; $\hat{y_i}$ = signal value at concentration i, calculated from the calibration curve; yi= signal at concentration i; n = number of different concentrations in the calibration curve; p = number of replicates for each different concentration in the calibration curve

Lack-of-Fit Test Ftab mathematic formulae

$$F_{tab} = INV.F.DIR\ (0{,}05;\ n-2;\ n(p-1))$$

Legend: n = number of different concentrations in the calibration curve; p = number of replicates for each different concentration in the calibration curve

Goodness-of-Fit Test Fcalc mathematic formulae

$$F_{calc} = \frac{MSSfactor}{MSS\ residuals} = \frac{\sum(\hat{y_i} - \bar{y_i})^2/(p-1)}{\sum(y_i - \hat{y_i})^2/(n-p)}$$

Legend: $\bar{y_i}$ = average signal value of the replicates at concentration i; $\hat{y_i}$ = signal value at concentration i, calculated from the calibration curve; yi= signal at concentration i; n = number of different concentrations on the calibration curve; p = number of replicates for each different concentration on the calibration curve

Goodness-of-Fit Test Ftab mathematic formulae

$$F_{tab} = INV.F.DIR\ (0{,}05;\ p - 1;\ n - p)$$

Legend: n = number of different concentrations in the calibration curve; p = number of replicates for each different concentration in the calibration curve
